# Prevalence of Quackery in America

**Published:** 1852-01

**Authors:** James H. Stuart

**Affiliations:** Erie, Pa.


### ARTICLE III.

*Prevalence of Quackery in America.* By JAMES H. STUART, A. M., M. D.

In glancing at our country as compared with others, we are compelled, however reluctantly, to admit that, though incomparably better in most respects, she is yet in some lamentably worse. Among these latter, rises pre-eminent the hydra head of quackery. In no other country has this body and soul destroying crime reached such awful and impudent power. Turn where you will, from the magnificence of the large city to the humility of the country village, quack advertisements, often of the most nauseous and disgusting kind, stare you full in the face. Broad sheets are printed and forced into your very houses, whose lightest word is sufficient to sully a pure soul. The young are informed of vices which their imagination never conceived of before. The most private and shocking diseases are shamelessly paraded; and fools of both sexses are found publishing confessions of having been cured of disorders, to even a knowledge of which, modest persons would never own. Mere boys and girls read these polluting developments with an avidity only equalled by their eagerness for that literature, which law has made it penal to circulate. Mock "physiologies," &c., are issued to delude the unwary, aud entrap them into those very vices which they pretend to defend them from.

Now why is all this? Why is it that a man cannot bring up his family in innocence and purity without the interference of these vile poison venders, some of whom rear palaces on the profits of their atrocious trade, and others send their children out with brazen face to associate on terms of equality with those of honorable lineage? Should not their origin render them forever outcasts; the "Pariahs of society," exiles to an eternal Coventry? Part of the cause of this is to be found in the progressive spirit of our country. As in all new countries, the go-ahead disposition still prevails to a great extent with us. Consequently, proper division of labor has not yet been established. Men grasp after the universal, and each individual seeks too anxiously to know and do a *little* of *everything*, ever to do any thing *well*. The result of this, is a deficiency, among other things, of *thoroughly educated* physicians, and a great superabundance of ignorant and conceited ones. Medical men are too much addicted to "making money," and care too little for the elevation of their profession. There are double of the men *nominally* in the profession than can ever obtain even a beggarly support from the respectable practice of it. Consequently, as no man will willingly starve, they direct their attention to other means of accumulating wealth, and neglect their professional cultivation, which at once opens a door for the real unmitigated quack to enter by. Or, st il worse, they prostitute the little medical knowledge they have to the vilest of purposes, issuing such advestisements as "Dr. ———, having directed his entire attention for some years to private diseases, challenges all cempetition," &c., or "Dr. ———'s celebrated cough medicine, the only one of any utility," &c.

O. W. Holmes has hit this off admirably. "Dr. C.

May be consulted for life's various ills,
And also sells the patent Pickwick pills;
Teeth are extracted. He will likewise vend
That well known ointment termed the loafer's friend."

I quote from memory. Perhaps there never was a period in the world's history, at which human credulity and absurd gullibility were so conspicuous as at present.

The history of delusions in past ages seems to have no effect whatever in suppressing credulity at present. People will laugh derisively at the advocates of the Cocklane ghost, and then turn with fond belief to the yet more absurd Rochester Rappings. They will wonder at the gullibility of those who had faith in Sir Kenelm Digby's vulnerary powders, and then with implicit confidence swallow some infinitesimal oyster shells to cure a brain fever. The efficacy of the "King's touch" for scrofula, excites their compassionate wonder, yet will they take cayenne pepper for acute gastritis. O humanity, humanity, how great, how small, how wise, how foolish thou art! All are willing to pull motes from a brother's eye.

"Oh wad some power the giftie gie us,

To see oursels as ithers see us."

Our country is essentially a money making one. Time is occupied in *accumulating* wealth, but very little in spending it. Let a man only *succeed* in his attempt to "gather gear by every wile," no matter whether "justified by honor" or not, and he is almost universally respected. Nothing is despised by which the great object is attained. Men are very anxious to punish a robber, for he takes *forcibly* from them their hard earned gains. But the quack steals so indirectly that they are scarce conscious of the theft. They consider his trade a business one, and regard their intercourse with him as a struggle of cunning. No one desires to suppress his vile trade by law, for each individual supposes himself sharp enough to escape from his clutches, and as each is successively outwitted, he maintains a discreet silence, lest he should be *known* for a fool. All the quack's *successes* are bruited abroad, because the one who speaks of them feeds his own vanity by showing the extraordinary intelligence which actuated him in employing the charlatan. His failures either occupy nameless graves, and "dead men tell no tales," or halt through life in sullen silence, for it would do no good to warn others, and human nature leads them to smile grimly, as one after another drops into the same trap which caught them. The leniency of the law is a great cause of quackery ; but I cannot consider that a decided evil, so long as innumerable medical schools send out hundreds of ignorant doctors annually, a diploma cannot be considered a proper criterion of ability. Hence any law discriminating *merely* between graduates and non-graduates would be unjust. A *radical* change must be made, or none at all. It is unfair to say to one man, you have not had means for a collegiate education, *therefare* you shall not practice ; and to another, You *were* able to buy a diploma, therefore you *shall*. Perhaps the main cause of quackery is the want of union among educated medical men. Instead of joining together to suppress all irregular practice they are much more apt to be engaged in decrying each other, either directly or by knowing looks and inuendoes.

People soon remark this, and naturally think, if these men have not confidence in each other, how can *we* confide in them? They are distracted how to decide among so many conflicting interests, and too often betake themselves to quackery for relief, as the soul tossed about in the sea of polemic theology, too often seeks rest in the dark abysses of infidelity. There is also a want of moral honesty too prevalent in the profession. Physicians will resort almost unconsciously, themselves, to petty tricks, which a moment's reflection would assure them are highly reprehensible. For instance, they will assume infallibility, and pronounce positive opinions on subjects which it is really impossible to be certain. These opinions, of course, often prove fallacious, and the community, deceived and disgusted, turn to the mountebank, who promises the most ridiculous things, with a feeling that they are not *much* more to be cheated by him than by educated men. Medical organization has done much, and, let us hope will yet do more for the suppression of quackery ; but is not as efficacious as it might, and ought to be. For the prevalent opinion regarding it is that it is undertaken, not for the good of the community, but for the private advantage of the individuals associated together, and to put down certain others whose interests are inimical to theirs. And no pains is taken by us to change this opinion. No means are used to convince the laity, that scientific physicians unite, *not* to put down, but to build up, not for the further degradation of those who are too low already, but merely to separate from, and declare their non-identity with them. We think these few causes are the potent ones of quackery. Now what can be done to effect a cure? Were it possible, it would be good experiment for all respectable physicians to cease practice entirely for a year or two. The massacres committed by quacks would then be fully evident to every body, and many would undoubtedly expatiate their crimes by lynch law, victims to the rage of those whose friends they had murdered. But on the return of educated men to practice, a new race would of course arise, perhaps worse than the former. If the whole community could be well educated, it would suppress quackery. But that is impossible. I see no other way, then, but quietly and firmly to continue the great work of reformation in the slow and sure course it is now taking; to improve ourselves, to increase the stringency ef our organization, and then, having thus done our duty, to leave the responsibility of their own actions with the people themselves. Those who have sense enough "to discern the evil from the good," will profit by their discrimination, and it will not be *our* fault, if those who have not, *die* in their ignorance, perhaps after having been beggared by their gullibility, to have inscribed for their epitaph,

"—— He died a codger powny's death,
At some dike side."

[*N. J. Med. Reporter.*

ERIE, PA., Nov. 1851.

